# Rest, shade, hydration and hygiene for the prevention of kidney injuries and inflammation in a Nicaraguan sugarcane worker cohort

**DOI:** 10.1136/oemed-2025-110128

**Published:** 2025-08-12

**Authors:** Erik Hansson, Jason Glaser, Ilana Weiss, Esteban Arias-Monge, Felipe Pacheco-Zenteno, Nathan H Raines, Michael Silva-Peñaherrera, Javier Vasquez, Zoey E Castellón, Scarlette Poveda, Fatima I Cerda-Granados, William Martinez-Cuadra, Denis Chavarria, Rebekah A I Lucas, Ulf Ekström, Kristina Jakobsson, Catharina Wesseling, David H Wegman

**Affiliations:** 1 La Isla Network, Washington, District of Columbia, USA; 2 School of Public Health and Community Medicine, Institute of Medicine, Sahlgrenska Academy, University of Gothenburg, Gothenburg, Sweden; 3 Division of Nephrology, Department of Medicine, Beth Israel Deaconess Medical Center, Harvard Medical School, Boston, Massachusetts, USA; 4 Occupational Health Management, Ingenio San Antonio/Nicaragua Sugar Estates Limited, Chichigalpa, Nicaragua; 5 School of Sport Exercise and Rehabilitation Sciences, University of Birmingham, Birmingham, UK; 6 Department of Laboratory Medicine, Lund University, Lund, Sweden; 7 University of Massachusetts Lowell, Lowell, Massachusetts, USA

**Keywords:** Workers, Kidney Diseases, Occupational Health, Workload, Climate

## Abstract

**Objectives:**

To study the effect of a progressively enhanced rest–shade–hydration–hygiene (RSHH) intervention on kidney injury and inflammation biomarkers, and rates of clinical acute kidney injury (AKI) in Nicaraguan sugarcane workers with a very high rate of chronic kidney disease of non-traditional origin (CKDnt).

**Methods:**

We analysed serum creatinine and C-reactive protein (CRP) and leukocyturia from samples obtained before and at the end of four harvest seasons (H1–4). An increase in creatinine≥0.30 mg/dL over the harvest was considered incident kidney injury (IKI). Rates of clinically diagnosed AKI were obtained from medical records. Each season the RSHH intervention included progressively longer and more frequent rest periods with improved access to shade and hydration, implementation monitoring, qualitative interviews and health outcome assessments.

**Results:**

1044 workers were followed for 1938 person-harvests. Among burned cane cutters, the job group with the highest workload and worst outcomes initially, there were decreasing rates of IKI (21% in H1 to 1% in H4, p<0.01), AKI (20/1000 worker-months to 8/1000 worker-months, p<0.01) and end-harvest leukocyturia (26% to 1%, p<0.01), and less rise in cross-harvest CRP (median 1.75-fold increase in H1 to 1.00 in H4, p<0.01).

**Conclusion:**

Kidney outcomes among outdoor heat-stressed workers at high risk of CKDnt improved as a structured RSHH intervention was implemented and committed to by workplace management. The findings support a causal relationship between occupational heat stress, kidney injury and CKDnt and point to possibilities for prevention.

**WHAT IS ALREADY KNOWN ON THIS TOPIC**

- Heat-stressed workers in Mesoamerica are at a very high risk of kidney injuries and chronic kidney disease of non-traditional origin (CKDnt). Initial intervention studies have reported that improved rest–shade–hydration practices appear to prevent kidney injuries.

**WHAT THIS STUDY ADDS**

- This cohort is the hitherto largest rest–shade–hydration–hygiene (RSHH) intervention study conducted in a working population at risk of CKDnt, and the first to show its preventive impact on preserved kidney function, decreased markers of systemic and kidney inflammation and decreased rates of clinical acute kidney injury.

**HOW THIS STUDY MIGHT AFFECT RESEARCH, PRACTICE OR POLICY**

- This study provides additional support to a causal relationship between heat stress, kidney injuries and CKDnt among Mesoamerican workers by identifying large declines in kidney injury rates and improvements in biomarkers during a structured RSHH intervention. Applying these interventions on a larger scale can help prevent the occupational heat stress-related kidney injury contributing to CKDnt and will likely be an important tool in other climate change-affected populations.

## Introduction

 Sugarcane workers are at a high risk of chronic kidney disease of non-traditional origin (CKDnt), a nephropathy arising among labourers working strenuously in hot climates that has caused tens of thousands of deaths in Mesoamerica over recent decades.^1 2^ Heat-stressed workers in Mesoamerica have high rates of acute kidney injury (AKI), especially following intense work, such as sugarcane harvesting.^3^,^5^ Workers performing physically strenuous jobs (such as sugarcane cutters) and generating substantial metabolic heat as a result have much higher risk of kidney injury than those performing less strenuous jobs (such as irrigation repair workers (IRWs) and supervisors).^6^

A leading hypothesis about the pathophysiology of CKDnt is that chronic disease occurs because of repeated AKI with incomplete subsequent recovery, ultimately leading to permanent loss of kidney function. Previous studies have described this pattern among sugarcane workers, whose AKI events have been linked to occupational heat exposure.^36^,^8^ Inflammation, measured by C-reactive protein (CRP)^9^ and leukocyturia,^10 11^ often co-occurs with reduced kidney function in these workers.

Despite several observational studies linking occupational heat stress to CKDnt in Mesoamerica,^1^ there have only been a few studies^7 12 13^ reporting on rest–shade–hydration–hygiene (RSHH) interventions on kidney injury outcomes. An RSHH programme is a structured intervention designed to mitigate the effects of heat stress among workers. This approach incorporates mandatory rest breaks, access to shaded areas, adequate hydration facilities and hygiene measures such as proper latrine availability. The intervention also emphasises monitoring adherence and the integration of health practices and evaluation of health outcomes into workplace routines. This study aimed to assess whether biochemical indicators of kidney injury, and kidney and systemic inflammation, as well as rates of clinically detected AKI improved as an RSHH intervention was gradually enhanced over 4 years among Nicaraguan sugarcane workers.

## Methods

### Setting and study population

Ingenio San Antonio (ISA) is a sugarcane mill in northwestern Nicaragua, a CKDnt hotspot.^2^ The sugarcane industry is a main employer in the area, mostly hiring workers for the 6-month harvest season from approximately November to April. Individuals were eligible to perform sugarcane-related work (manual and supervisory work) at ISA if they had a serum creatinine (sCr)<1.3 mg/dL for men and <1.0 mg/dL for women up until the 2018/2019 harvest. From the 2019/2020 harvest onwards, this cut-off was replaced by estimated glomerular filtration rate (eGFR) of >90 mL/min/1.73 m^2^ for both men and women.

Workers experiencing illness during the harvest may receive care at the mill’s hospital.^3 5^ At this hospital, sCr levels are routinely measured in workers presenting with relevant medical symptoms. However, rather than the standard criteria for AKI of a 0.3 mg/dL increase from baseline values over 48 hours,^14^ an sCr≥1.3 and 1.0 mg/dL cut-off of in men and women respectively was used.

Burned cane cutters (BCCs) and seed cutters (SCs) perform the highest-intensity work, while drip IRWs perform moderate-intensity work and field support staff (FSS) overseeing these groups perform low-intensity work.^15^ More specifically, BCCs cut mature cane after it had been burned in the field in the preceding day or evening. SCs harvest green, living cane and cut it into smaller pieces which are then used as seedlings. Both cutter groups are paid by the piece. The work tasks of IRWs consist of inspecting recently harvested cane fields for underground irrigation tube leaks, digging a few decimetres to identify these leaks, and then repairing the tubes using tape. IRW are not piece-paid.

### Data collection

#### Adelante/PREP cohort: biochemical indicators

Among the ISA workforce, all employed sugarcane cutters (BCCs and SCs), and two out of four IRW groups, and all FSS overseeing the recruited groups of BCCs, SCs and IRWs were recruited before the harvest that started in November 2017 (harvest 2017–2018, henceforth ‘H1’). These workers were sampled in parallel with the mill’s pre-employment screening. At the end-harvest, all workers present at work were sampled, but biological measurements could not be performed on those who were not present at the end-harvest.

Pre-harvest and end-harvest sampling continued during the 2018–2019, 2019–2020 and 2020–2021 harvests (henceforth ‘H2’, ‘H3’ and ‘H4’, respectively) within these groups, although the COVID-19 pandemic caused additional logistical challenges at the end of H3 and during H4.

Leukocyturia was assessed by manual reading of a Combur 10 dipstick in fresh urine samples at the ISA mill hospital. All serum samples were analysed for sCr at the mill hospital laboratory on the day of collection, so that results could be returned to the participants in a timely manner. However, these values were not used for our analysis. To ensure high-quality batchwise analysis, we instead analysed sCr and CRP concentrations in samples that had been frozen within 4 hours of collection in the field and subsequently shipped to the analysis site, where they were stored at −80°C. Both pre-harvest and end-harvest samples from each harvest were thawed and analysed in a single batch to eliminate batch effects otherwise potentially biasing cross-harvest comparison. In the first two harvests, serum samples were analysed for sCr and CRP at the Clinical Chemistry Department at Lund University using a Cobas 701 instrument (Roche). For the second two harvests, sCr and CRP were analysed using a Cobas 6000 instrument (Roche) at Lab San José, Costa Rica. All pre-harvest and end-harvest samples from the same worker were analysed on the same machine and in the same batch.

#### ISA mill hospital records: clinical AKI events

We accessed clinical reports of workers within all BCCs, SCs and IRWs groups fulfilling the mill definition of AKI starting from January 2018. Records were constructed by an occupational health and safety (OHS) nurse who abstracted predefined clinical data into an Epi-Info dataset, primarily intended to be used by the mill OHS department. Information on the job title of workers with AKI was identified by linking human resource (HR) department data to the company ID of workers diagnosed with AKI. Denominators, the number of workers employed in each work group of BCCs, SCs and IRWs, were obtained by month from the HR department, and, therefore, these data differed from the numbers included in the cohort study. Identifying FSS belonging to work groups participating in the cohort study was impossible and that work group was therefore not included in this analysis. Workers dropping out of the cohort study and not providing end-harvest samples were included in this analysis nevertheless.

### Intervention design and implementation assessment

In 2010, sugarcane cutters at ISA did not have mandated breaks.^16^ Workers were allowed to bring their own water to work, but there was also additional water available.^16^ Mandatory 20 min breaks at 08:00 and 10:00 for BCCs were instituted in 2013, and then changed to 10–20 min breaks every hour from 09:00 in 2016. Efforts to strengthen the intervention and systematically evaluate the results increased with the launch of the Adelante Initiative intervention study (launched in 2017–2018; https://adelanteinitiative.org/), and subsequently the Prevention, Resilience, Effectiveness and Protection (PREP) study (launched in 2019–2020; https://laislanetwork.org/programs/).

During the first year of Adelante (H1), existing heat stress practices were observed, and factors which could have influenced their implementation were recorded, but no input from the researchers was provided on improvements in practices during the harvest. Practices included health promoters who oversee workers’ hydration and provide training on heat stress prevention and other health-related topics during rest breaks.

**Figure 1:**
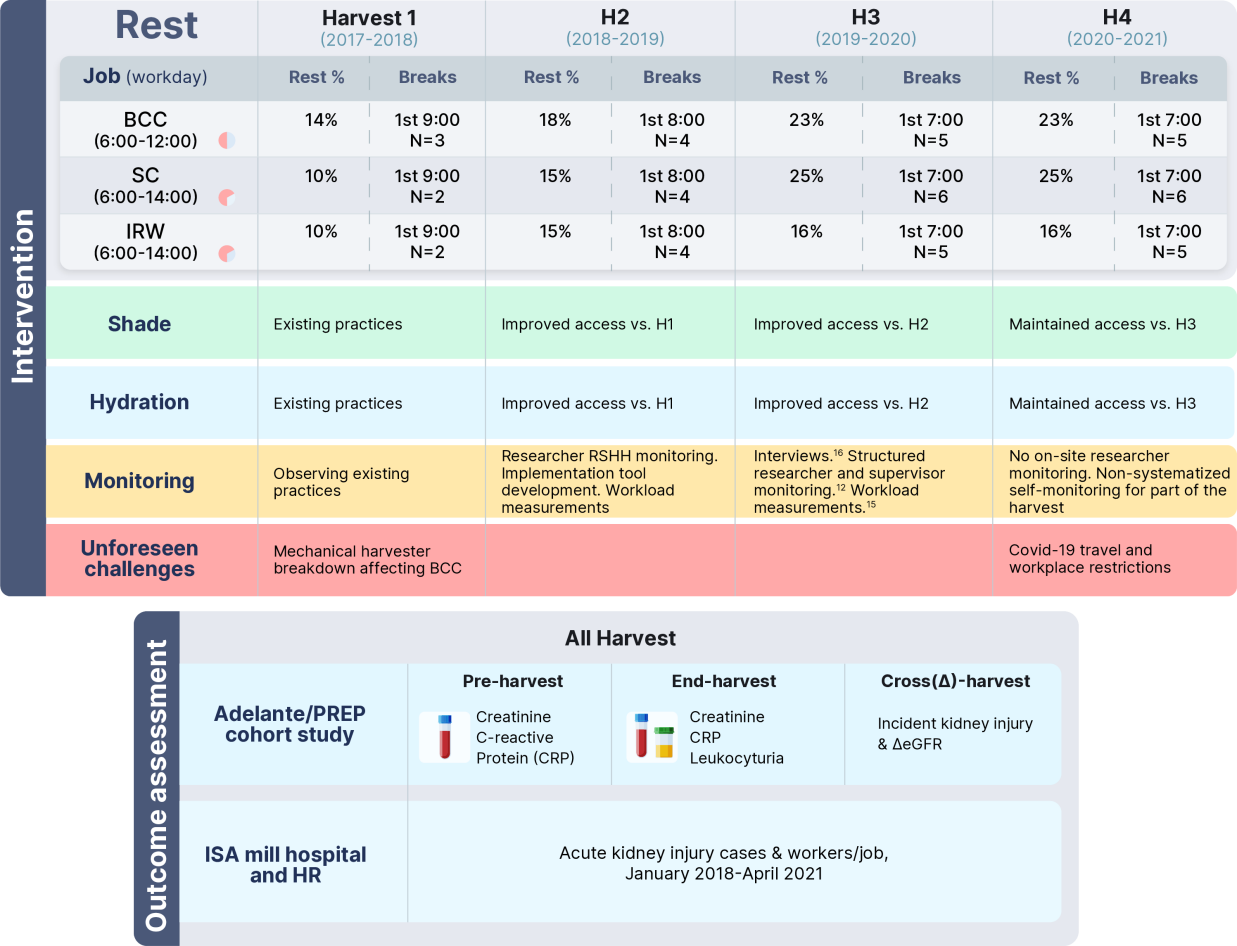
Study overview. BCC, burned cane cutters; eGFR, estimated glomerular filtration rate; HR, human resource; IKI, incident kidney injury, an increase in serum creatinine by 0.3 mg/dL across the harvest season; IRW, irrigation repair workers; ISA, Ingenio San Antonio; PREP, Prevention, Resilience, Effectiveness and Protection; Rest %, rest time as % of total time worked in a day; RSHH, rest–shade–hydration–hygiene; SC, seed cutters.

At the start of the second harvest (H2), work–rest schedules were enhanced and restructured^7 12^ (figure 1) to provide more regular rest periods, longer overall rest, as well as an earlier rest period. All modifications of the heat stress programme were developed together with the mill OHS organisation. It was emphasised that rest schedules were mandatory, and supervisors reported an increased commitment to enforcing rest breaks.^17^ Mandatory breaks are important for piece-paid workers’ adherence to breaks.^18^ Recommendations on how to distribute fluid and guarantee access to shade were provided. This included the acquisition of more shade tents to ensure that workers could access a tent nearby and avoid crowding as this may hinder effective heat transfer by ventilation. The importance of having adequate latrines available to the workers was stressed. Observations of intervention implementation quality, including assessments of the availability and use of shade tents, hydration and latrines, and the adherence to rest schedules were performed by research staff.^12^ It was identified that the implementation quality varied substantially between the SC groups and that this was related to kidney health outcomes.^7 12^

During the third year (H3), work–rest schedules were further enhanced by increasing the overall rest proportion and including an earlier rest break (table 1).^12^ Heart rate measurements performed in cutters in H2 and H3 documented lower physiological strain among BCC following this enhanced rest schedule.^15^ Continued efforts were made to monitor intervention implementation and adherence by developing and using structured semi-quantitative self-monitoring tools for field supervisors, which were supplemented by research staff field visits.^12^ Qualitative interviews from an organisational psychology perspective were conducted with various levels of the mill management hierarchy to explore factors enabling and hindering implementation of the RSHH intervention.^17^ In addition to revealing a progressive increase in management’s commitment, including supervisory field staff, as well as cultural and policy changes aimed at integrating health prevention into production processes, the findings prompted the mill to address the identified gaps.

**Table 1:** Kidney-related biomarkers during the study period among male sugarcane cutters

Harvest	Burned cane cutters				Seed cutters			
	1	2	3	4	1	2	3	4
Participants with pre-harvest sCr (n)	189	235	173	188	156	245	218	266
Participants without cross-harvest sCr (n)	61	67	13	24	50	115	83	77
Age, years, mean (SD)	31 (9)	31 (8)	29 (7)	33 (7)	26 (6)	30 (7)	27 (7)	29 (6)
eGFR_pre-harvest_, mL/min/1.73 m^2^, mean (SD)	90 (25)	88 (24)	101 (32)	97 (20)	105 (26)	98 (27)	110 (17)	98 (26)
CRP_pre-harvest_, median (IQR)	1.5 (0.7–2.7)	1.4 (0.8–4.2)	0.7 (0.7–2.1)	1.3 (0.7–3.2)	1.6 (0.7–3.2)	0.9 (0.7–2.1)	1.0 (0.7–2.0)	1.5 (0.7–3.0)
Participants with cross-harvest sCr (n)	128	168	160	164	106	130	135	189
Age, years, mean (SD)	32 (9)	32 (8)	32 (8)	33 (8)	27 (6)	27 (6)	27 (7)	28 (7)
eGFR_pre-harvest_, mL/min/1.73 m^2^, mean (SD)	96 (20)	100 (17)	100 (18)	100 (18)	102 (24)	102 (24)	104 (18)	103 (18)
CRP_pre-harvest_, median (IQR)	1.0 (0.7–2.4)	1.0 (0.7–2.2)	0.7 (0.7–3.1)	0.7 (0.7–2.0)	1.2 (0.7–2.3)	1.1 (0.7–2.6)	1.1 (0.7–2.3)	0.7 (0.7–1.9)
IKI, N (%)	27 (21%)	9 (5%)	2 (1%)	2 (1%)	5 (5%)	12 (9%)	2 (1%)	4 (2%)
ΔeGFR mL/min/1.73 m^2^, median (IQR)	−4 (−18, 1)	−1 (−8, 4)	2 (−2, 7)	0 (-5, 5)	−3 (−9, 1)	−1 (−6, 3)	5 (−1, 11)	−1 (−8, 3)
ΔCRP, median (IQR)	1.75 (1.04–5.56)	1.00 (0.81–1.84)	1.00 (0.73–1.84)	1.00 (0.95–2.04)	1.03 (0.69–2.21)	1.27 (0.93– 2.83)	1.00 (0.59–1.84)	1.23 (1.00–2.16)
Leukocyturia_end-harvest_ (any +) (%)	23/90 (26%)	1/168 (1%)	2/127 (2%)	1/163 (1%)	16/106 (15%)	7/127 (6%)	1/131 (1%)	3/180 (2%)

CRP, C-reactive protein; eGFR, estimated glomerular filtration rate; IKI, incident kidney injury; sCr, serum creatinine.

By H4, the COVID-19 pandemic had reached the mill. The company completely restricted outsider visits. Information from ISA indicates that sugarcane harvesting continued as planned. Efforts to reduce COVID-19 transmission (physical distancing while commuting and during rest, encouraging hand hygiene and face mask use, as well as pre-shift fever screening) were introduced. Researcher-led efforts to improve intervention design and monitor intervention implementation were cancelled, and the self-monitoring tool was used intermittently by mill staff as focus was on COVID-19 prevention. No attempts to enhance the intervention were made between H3 and H4.

### Statistical analyses

#### Adelante/PREP cohort: biochemical indicators

Temporal changes in kidney injury markers were assessed by job group and sex as the intervention effects may have been differentially well achieved by work groups and responses for these indicators may differ between men and women. Incident kidney injury (IKI) was defined as an increase in sCr of ≥0.30 mg/dL across the harvest season.^6^ Changes in eGFR (ΔeGFR, with negative values indicating loss of kidney function) across the harvest season were estimated by subtracting the pre-harvest from the end-harvest estimate. eGFR was estimated using the 2021 Chronic Kidney Disease Epidemiology Collaboration (CKD-EPI) equation.^19^

The change in CRP across the harvest season (ΔCRP, with positive values indicating increasing systemic inflammation) was calculated as the end-harvest CRP concentration divided by the pre-harvest concentration after replacing values below limit of detection (LOD, 0.6 mg/L in H1–H2 and 1.0 mg/L in H3–H4) by LOD/√2, and thereafter transformed using the natural log as this was highly skewed.

Any leukocyturia (≥+) at the end of harvest was considered a positive kidney or urinary tract inflammation marker.

The proportion of workers with IKI and leukocyturia was analysed using mixed-effects Poisson regression, and ΔCRP and ΔeGFR were analysed using mixed-effects linear regression. Each individual had random intercepts. A linear trend for harvest year was added into the models to assess whether there had been a trend in biomarker results during the study period. Models were adjusted for first pre-harvest eGFR and age.


(1) $$outcome=\begin{array}{l} \beta_{l}\times harvest\;year+\beta_{2}\times age+\beta_{3}\times \;eGFR_{pre-harvest}\\ \;+individual\;intercept+mean \end{array}$$

Equation 1 describes the regression models used for estimating trends in biomarker outcomes.

We also considered a non-linear parameterisation of harvest year (online supplemental table 4).

Only workers with biomarker data from before and after a harvest were included in analyses. Workers could be included in only one or several of the harvest seasons.

#### ISA mill hospital records: clinical AKI events

Trends in the AKI incidence rate during harvest season over the intervention period were examined using Poisson regression. We restricted the period of comparison to January–April as the harvest season spans November–April and there were no data available for November and December 2017.

## Results

A total of 1044 workers in the Adelante/PREP cohort were followed for 1938 completed harvest seasons. Congruent with the overall workforce at ISA, most of the workers were male, and 95% were younger than 45 years old (table 1 and online supplemental tables 1 and 2).

Among male cutters, the risk of IKI and leukocyturia, as indicative of kidney injury and inflammation, decreased during the study period (table 2). Mirroring this reduction in kidney injury rates, the pronounced drops in eGFR across initial harvests among cutters improved during the study period. The biomarker of systemic inflammation, ΔCRP over the harvest, decreased among BCCs (all p<0.05 for a linear improving trend) (table 2). In H1, median end-harvest CRP levels among BCCs were almost at 5 mg/L, decreasing to approximately 1 mg/L in later years. Male IRWs and FSS started with better results for these biomarkers before the enhanced intervention was implemented and exhibited no clear trends over time (online supplemental table 1).

**Table 2:** Regression coefficients for temporal trends in kidney-related biomarkers among male workers

Outcome, effect estimate unit	Incident kidney injury, incidence ratio (95% CI) (p)	ΔeGFR, mL/min/1.73 m^2^ (95% CI) (p)	ΔCRP, mg/dL (95% CI) (p)	Leukocyturia_end-harvest_ (any +), incidence ratio (95% CI) (p)
Job group				
Burned cane cutters	0.33 (0.21 to 0.50) (<0.01)	3.2 (2.2 to 4.1) (<0.01)	−0.2 (−0.2 to –0.1) (<0.01)	0.17 (0.08 to 0.33) (<0.01)
Seed cutters	0.70 (0.48 to 1.01) (0.06)	1.3 (0.3 to 2.3) (0.01)	0.0 (−0.1 to 0.1) (0.51)	0.43 (0.28 to 0.66) (<0.01)
Irrigation repair workers and field support staff	Too few events	−0.4 (−1.4 to 0.6) (0.47)	−0.1 (−0.2 to 0.0) (0.20)	Too few events

CRP, C-reactive protein; eGFR, estimated glomerular filtration rate.

There were fewer female workers performing strenuous work and, except for leukocyturia, biochemical indicators were generally better than for male workers (online supplemental table 2). End-harvest leukocyturia prevalence decreased among female SCs (online supplemental table 3).

**Figure 2:**
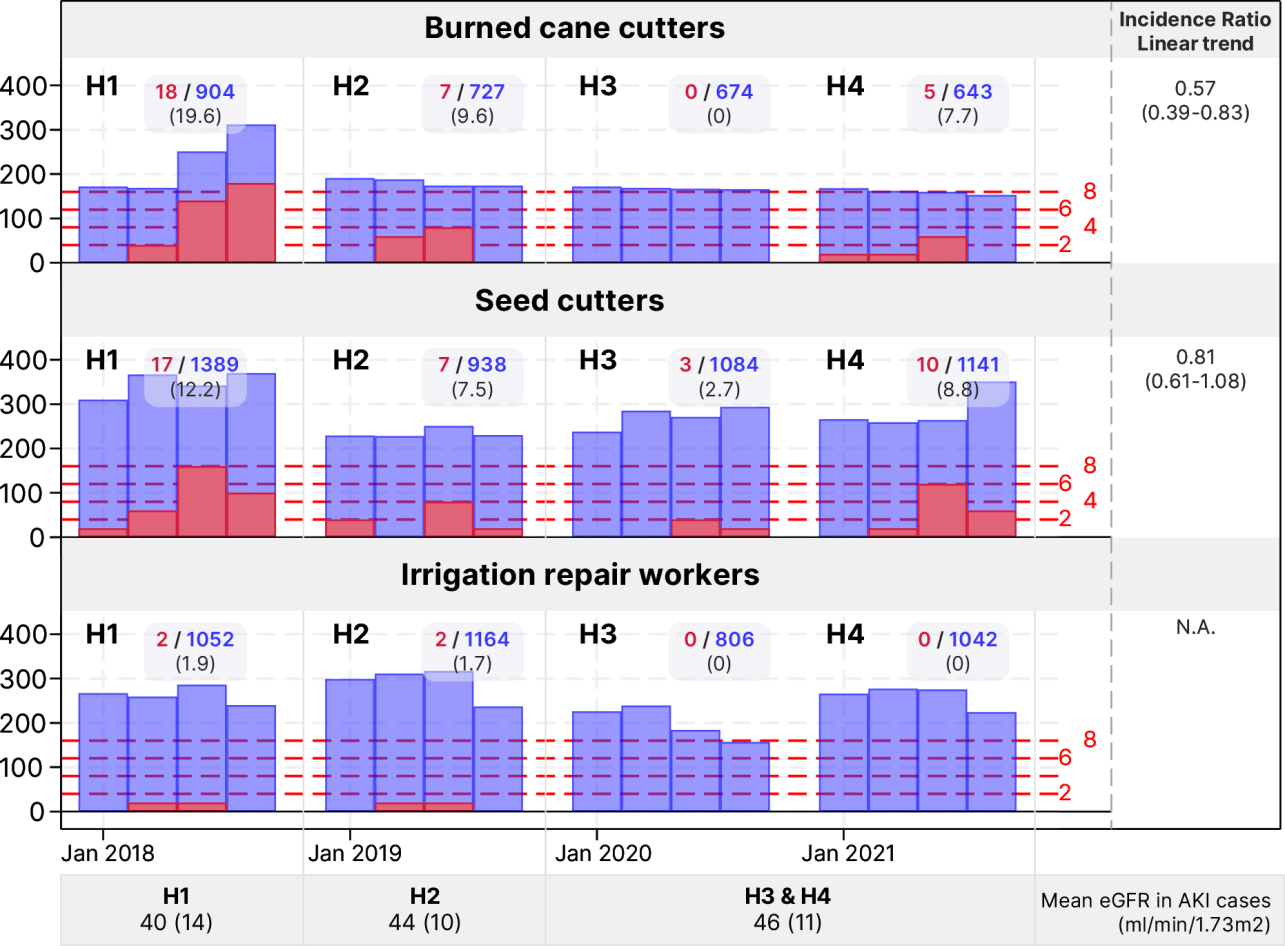
Number of worker-months and clinical acute kidney injury (AKI) rate by harvest and work group. Light-blue bars represent the number of worker-months and red dots and black lines clinical AKI rates. eGFR, estimated glomerular filtration rate.

There were 77 cases of clinical AKI among the studied job groups, 71 of which occurred in January–April (online supplemental figure 1). The AKI rate had a statistically significant decreasing trend among BCCs (total number of cases=32), was in the direction towards improvement in SCs (n=41) and remained low in IRWs (n=4) (figure 2).

## Discussion

### Key findings

During implementation of a structured RSHH intervention, now followed over the course of 4 years, kidney injury rates and kidney and systemic inflammation markers dropped in male workers at initial high risk. To our knowledge, this study reports on the largest and longest running RSHH intervention so far, and the findings provide actionable evidence highly relevant for a warmer future.

### Strengths and limitations

The study design was a pragmatic open-label intervention as randomisation would have been practically and ethically impossible. Comparisons were made to preceding years rather than parallel control groups. While this design adds to ecological validity and generalisability of findings, it means there is a possibility that unmeasured confounders or an unknown strong calendar year effect affected the results. Environmental temperatures were similarly high throughout the four harvest seasons.^3^ The particularly poor outcomes for BCC in H1 were related to a very stressful harvest with the breakdown of several mechanical harvesters and consequent poor adherence to existing heat prevention measures. It also means that the effect seen here may not only be due to specific elements of the intervention design, such as exact duration or number of rest breaks, but rather due to an increased adherence to existing workplace occupational safety and health (OSH) policy. Still this observation emphasises the need for well-designed, implemented and resilient RSHH interventions. There was no formal worker representation; however, worker experiences and insights were regularly gathered informally while collecting biological and questionnaire information.

Aside from progressive enhancements of the RSHH intervention, the change in employment criteria (introduction of an eGFR minimum in place of an sCr maximum required for hire between H2 and H3) may have contributed to decreased levels of kidney injury and systemic inflammation during the harvest season by better excluding workers with established early CKDnt. However, considering (a) the improving trend persisted after eGFR_pre-harvest_ adjustment (table 2), (b) average eGFR_pre-harvest_ improved only marginally from H2 to H3 (table 1) and (c) improvement occurred before employment criteria changed (ie, from H1 to H2) (table 1), we consider this an unlikely main explanation for the trends seen. Including the same workers each year would have minimised risk of bias and maximised statistical power, but a substantial proportion did not work every harvest.

As previously reported,^6 7^ a large proportion of workers left work due to AKI in H1 and H2. 39% and 31% of the cutter drop-outs who were reached at home after H1 and H2 respectively reported having been on sick leave for AKI.^6 7^ At-home follow-up of workers not attending end-harvest sampling was cancelled after H3 due to the start of the COVID-19 pandemic. When it resumed after H4, 4 out of 30 (13%) BCCs and SCs interviewed afterwards reported having been on sick leave for AKI during the harvest season. Considering this decrease in proportion of drop-outs reporting kidney injury, it is unlikely that differential changes in follow-up of unwell workers led to the improved biomarker outcomes seen here, but lack of information on all non-returning workers introduces uncertainty.

Intervention effects were primarily assumed to have a constant gradual trend during the intervention period rather than a binary no-intervention/intervention effect or a categorical year-by-year effect. This is not necessarily the best option for all similar studies, but we chose this approach to limit the number of model parameters and as the intervention was gradually enhanced throughout H1–H3. The findings using a non-linear parameterisation of year suggested some non-linearity (online supplemental table 4), which may have been due to the impact of COVID-19 in H4. The regression coefficients reported serve to estimate the impact of this intervention at this specific worksite at this time and place rather than generalisable effect estimates.

**Figure 3:**
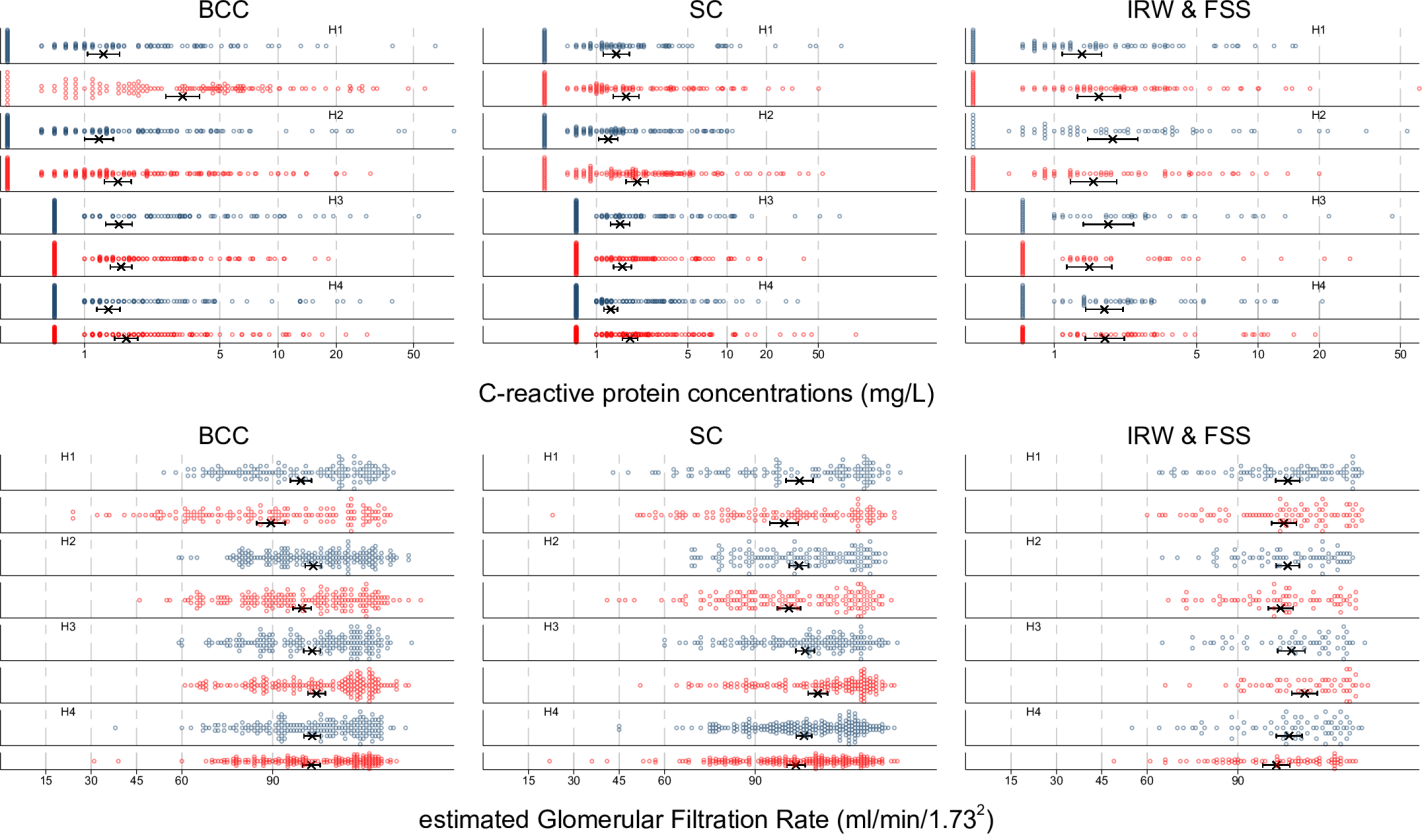
Distribution of markers of systemic inflammation (CRP) and kidney function (eGFR) before (blue) and at the end (red) of harvests 1–4 among male workers. X and bar denote mean with 95% CI. Graphs include only workers starting and finishing each harvest. A 90 mL/min/1.73 m^2^ pre-employment cut-off was started in B3. However, this hiring decision was based on results from the local lab (and not the external results as presented here). Further, hiring decisions were made after re-sampling workers with low eGFR at the initial measurement shown here. BCC, burned cane cutter; CRP, C-reactive protein; eGFR, estimated glomerular filtration rate; FSS, field support staff; IRW, irrigation repair worker; SC seed cutter.

Clinically apparent AKI had a slightly increasing trend between H3 and H4 (figure 2). This may be explained by the inclusion of less severe AKI cases due to more active efforts to identify those experiencing ill health at the mill during the observation period,^3^ a development which likely benefits the workers. Higher eGFR among AKI cases in the subpopulations studied here supports that such a drift occurred (figure 3). Also, the COVID-19 pandemic in H4 may have led to more workers seeking hospital care with symptoms overlapping with AKI, some of whom may also have had an sCr elevation from COVID-19 or a combination of work in heat and COVID-19.

The consistent decreasing trend in the incidence and severity of all indicators, reflecting different biological processes, using different laboratory approaches and different catchment strategies, reduces the likelihood that the observed improvements in the groups at high initial risk are chance findings. A strong correlation between sCr and cystatin C across the harvest season indicates that the sCr-based outcomes reflect reduced renal function rather than merely changes in creatinine synthesis.^20^ Group-level eGFR or sCr changes across a few months are sensitive to drift of the analysis levels, necessitating −80° storage with careful mixing of thawed samples^21^ before batch analysis. Biomarkers such as CRP^9^ and leukocyturia^10 11^ may function as alternative intermediary outcomes to complement sCr changes.

### Interpretation

Previous research indicates that kidney injury during heavy work in heat predisposes workers to rapid eGFR losses.^3 4 7 8 22 23^ Our findings indicate that structured RSHH interventions can decrease kidney injury rates, consistent with a causal relationship between occupational heat stress, kidney injuries and ultimately CKDnt. This agrees with previous evidence from laboratory studies of human and animal models,^24^,^26^ one previous intervention study in sugarcane workers,^13^ a genome-wide association study^27^ and a large body of observational studies.^1 28^

Considering this evidence and global warming, there is a need to enhance intervention research and action to prevent occupational heat stress and preserve renal health. At the company level, productivity and economic evaluations indicate that this can be done without productivity losses and with a positive return on investments.^29 30^

The collaboration between researchers and mill departments during the iterative process of evaluating existing practices, improving protocols and monitoring of implementation, workload and outcomes was essential for the intervention. As indicated by interviews performed among supervisors and management, the collaboration between researchers and mill departments, the increased focus on health prevention and communication of health outcomes back to the mill workforce and management during the study may have contributed to shifting organisational priorities to further considering health metrics and outcomes in organisational policies.^17^

## Conclusion

Efforts to prevent heat stress via an RSHH intervention were associated with an improvement in biomarkers of kidney injury and systemic inflammation as well as reduced rates of clinically detected AKI events. Considering the link between kidney injury and CKDnt, such interventions are likely to prevent CKDnt onset or decrease its progression. Reducing occupational heat stress should be in focus for efforts to halt the CKDnt epidemic. Governments and regulatory agencies should continue efforts to implement and evaluate heat stress prevention programmes and strengthen their occupational health capacity.^31 32^ At a global level, our findings stress the need to mitigate climate change to preserve worker health and livelihoods.^33^

## Supplementary material

10.1136/oemed-2025-110128 online supplemental file 1

## Data Availability

Data are available upon reasonable request.
