# Supplementary material for: Rest, shade, hydration and hygiene for the prevention of kidney injuries and inflammation in a Nicaraguan sugarcane worker cohort
Source: Occup Environ Med. 2025 Aug 12;82(6):e110128. doi: 10.1136/oemed-2025-110128 (PMC12421119; doi:10.1136/oemed-2025-110128)
Supplement: online supplemental file 1 [file oemed-82-6-s001.docx]

Supplement Table 1. Biomarker results in male irrigation repair workers.

| Harvest | 1 | 2 | 3 | 4 |
| --- | --- | --- | --- | --- |
| Participants with pre-harvest SCr (N) | 109 | 112 | 121 | 91 |
| Participants without cross-harvest SCr (N) | 27 | 45 | 67 | 21 |
| Age, years, mean (SD) | 31 (9) | 28 (7) | 31 (8) | 31 (6) |
| eGFR_pre-harvest_, ml/min/1.73m^2^, mean (SD) | 95 (25) | 100 (24) | 104 (17) | 104 (18) |
| CRP_pre-harvest_, median (IQR) | 1.5 (0.7-5.5) | 1.7 (0.7-2.9) | 1.3 (0.7-2.7) | 2.0 (0.7-3.1) |
| Participants with cross-harvest SCr (N) | 82 | 67 | 54 | 70 |
| Age, years, mean (SD) | 30 (8) | 30 (8) | 29 (9) | 31 (8) |
| eGFR_pre-harvest_, ml/min/1.73m^2^, mean (SD) | 104 (18) | 104 (15) | 105 (17) | 104 (18) |
| CRP_pre-harvest_, median (IQR) | 1.2 (0.7-2.7) | 1.8 (0.9-3.8) | 1.5 (0.7-2.9) | 1.6 (0.7-2.8) |
| IKI, N (%) | 0 (-) | 1 (1%) | 0 (-) | 3 (4%) |
| ΔeGFR ml/min/1.73 m^2^, median (IQR) | -1 (-5, 3) | 0 (-8, 4) | 3 (-1, 9) | -2 (-11, 2) |
| ΔCRP, median (IQR) | 1.00 (0.81-1.81) | 0.97 (0.59-1.31) | 1.00 (0.47-1.38) | 1.00 (0.71-1.14) |
| Leukocyturia_end-harvest_ (any +) (%) | 0/80 (-) | 0/34 (-) | 0/51 (-) | 0/58 (-) |

Supplement Table 2. Biomarker results in female workers

|  | Seed cutters | | | | Irrigation repair workers and field support staff | | | | |
| --- | --- | --- | --- | --- | --- | --- | --- | --- | --- |
| Harvest | 1 | 2 | 3 | 4 | 1 | 2 | 3 | 4 |  |
| Applicants, with pre-harvest SCr (N) | 61 | 81 | 54 | 45 | 83 | 113 | 133 | 116 |  |
| Participants without cross-harvest SCr (N) | 19 | 35 | 15 | 7 | 14 | 36 | 46 | 20 |  |
| Age, years, mean (SD) | 31 (7) | 31 (7) | 30 (6) | 29 (8) | 28 (7) | 29 (6) | 33 (8) | 32 (8) |  |
| eGFR_pre-harvest_, ml/min/1.73m^2^, mean (SD) | 113 (18) | 105 (18) | 114 (15) | 106 (15) | 111 (25) | 115 (16) | 110 (16) | 109 (20) |  |
| CRP_pre-harvest_, median (IQR) | 2.0 (1.3-4.1) | 1.9 (1.1-3.7) | 2.8 (0.7-3.1) | 2.1 (1.6-3.9) | 2.2 (0.9-8.0) | 2.8 (1.1-5.5) | 2.7 (1.4-6.9) | 2.7 (0.7-6.1) |  |
| Finishers, with cross-harvest SCr (N) | 42 | 46 | 39 | 38 | 69 | 77 | 87 | 96 |  |
| Age, years, mean (SD) | 33 (6) | 32 (8) | 33 (7) | 34 (7) | 31 (6) | 31 (7) | 30 (7) | 32 (7) |  |
| eGFR_pre-harvest_, ml/min/1.73m^2^, mean (SD) | 99 (22) | 105 (19) | 104 (18) | 104 (16) | 110 (15) | 108 (15) | 111 (16) | 110 (15) |  |
| CRP_pre-harvest_, median (IQR) | 3.7 (1.2-7.8) | 3.0 (1.0-7.0) | 2.7 (1.0-7.4) | 2.6 (1.1-5.5) | 2.3 (1.1-4.3) | 1.8 (0.8-5.0) | 3.6 (1.4-8.5) | 2.8 (1.2-5.3) |  |
| IKI, N (% of finishers) | 0 | 1 (2%) | 0 | 1 (2%) | 0 | 0 | 0 | 0 |  |
| ΔeGFR, median (IQR) | -3 (-10, 1) | 0 (6, 2) | -1 (8, 3) | -5 (-11, 0) | 0 (-3, 4) | -2 (-6,2) | -1 (-6,6) | -3 (7,1) |  |
| ΔCRP, median (IQR) | 0.96 (0.59-1.68) | 1.11 (0.80-2.45) | 1.00 (0.74-1.24) | 1.18 (0.88-1.91) | 0.94 (0.57-1.26) | 1.13 (0.75-1.60) | 0.67 (0.39-1.12) | 0.91 (0.54-1.05) |  |
| Leukocyturia_end-harvest_ (any +) (%) | 10/42 (24%) | 6/46 (13%) | 1/39 (3%) | 2/38 (5%) | 19/68 (28%) | 3/56 (5%) | 3/81 (4%) | 13/87 (15%) |  |

Supplement Table 3. Regression coefficients for temporal trends in kidney-related biomarkers among female workers

| Outcome, effect estimate unit | Incident kidney injury, Incidence ratio (95% C.I.) [p] | ΔeGFR, ml/min/1.73 m^2^ (95% C.I.)  [p] | ΔCRP, mg/dL (95% C.I.) [p] | Leukocyturia_end-harvest_ (any +), Incidence ratio (95% C.I.) [p] |
| --- | --- | --- | --- | --- |
| Job group |  |  |  |  |
| Seed cutters (SC) | Too few events | -0.2 (- 1.5,1.2) [0.76] | 0.1 (0.0,0.2) [0.25] | 0.51 (0.31,0.84) [0.01] |
| Irrigation repair workers (IRW) and field support staff (FSS) | Too few events | -0.0 (-0.1, 0.0) [0.32] | -0.6 (-1.4, 0.2) [0.15] | 0.75 (0.56, 0.99) [0.04] |

p-values are for evidence of a linear trend across the study period.

Supplement Table 4. Regression estimates using a categorical parameterisation for harvest year

|  | Harvest | Male workers | | | Female workers | |
| --- | --- | --- | --- | --- | --- | --- |
|  |  | Burned cane cutters | Seed cutters | Irrigation repair workers | Seed cutters | Irrigation repair workers |
| ΔeGFR, ml/min/1.73 m^2^ (95% C.I.) | 1 | ref | ref | ref | ref | ref |
|  | 2 | 6.5 (3.7, 9.3) | 1.3 (-1.8, 4.5) | -0.5 (-3.3, 2.3) | 3.3 (-0.5, 7.2) | -3.1 (-5.6, 0.6) |
|  | 3 | 11.3 (8.4, 14.2) | 9.6 (6.4, 12.8) | 6.0 (2.9, 9.2) | 3.6 (-0.4, 7.6) | -0.4 (-3.0, 2.1) |
|  | 4 | 9.6 (6.7, 12.4) | 2.7 (-0.3, 5.7) | -3.0 (-6.0, 0.0) | -0.8 (-4.9, 3.3) | -2.8 (-5.3, -0.4) |
| ΔCRP, mg/dL (95% C.I.) | 1 | ref | ref | ref | ref | ref |
|  | 2 | -0.6 (-0.9,-0.4) | 0.3 (0.0, 0.6) | -0.4 (-0.7, -0.0) | 0.2 (-0.1, 0.6) | 0.1 (-0.2, 0.4) |
|  | 3 | -0.8 (-1.0,-0.5) | -0.1 (-0.4, 0.2) | -0.4 (-0.7, -0.1) | -0.0 (-0.4, 0.3) | -0.2 (-0.5, 0.0) |
|  | 4 | -0.6 (-0.8,-0.3) | 0.2 (-0.0, 0.5) | -0.2 (-0.5, 0.1) | 0.3 (-0.0, 0.7) | -0.0 (-0.3, 0.2) |
| Incident kidney injury, Incidence ratio (95% C.I.) | 1 | ref | ref | ref | ref | ref |
|  | 2 | 0.29 (0.14-0.62) | 2.07 (0.72-5.88) | Too few events | Too few events | Too few events |
|  | 3 | 0.07 (0.02-0.29) | 0.34 (0.07-1.78) |  |  |  |
|  | 4 | 0.07 (0.02-0.29) | 0.51 (0.13-1.91) |  |  |  |
| Leukocyturia_end-harvest_ (any +), Incidence ratio (95% C.I.) | 1 | ref | ref | ref | ref | ref |
|  | 2 | 0.02 (0.003-0.18) | 0.41 (0.17-1.01) | Too few events | 0.53 (0.19-1.46) | 0.19 (0.06-0.65) |
|  | 3 | 0.06 (0.02-0.28) | 0.06 (0.01-0.45) |  | 0.11 (0.01-0.83) | 0.13 (0.04-0.45) |
|  | 4 | 0.03 (0.003-0.19) | 0.14 (0.04-0.48) |  | 0.22 (0.05-1.02) | 0.53 (0.26-1.08) |


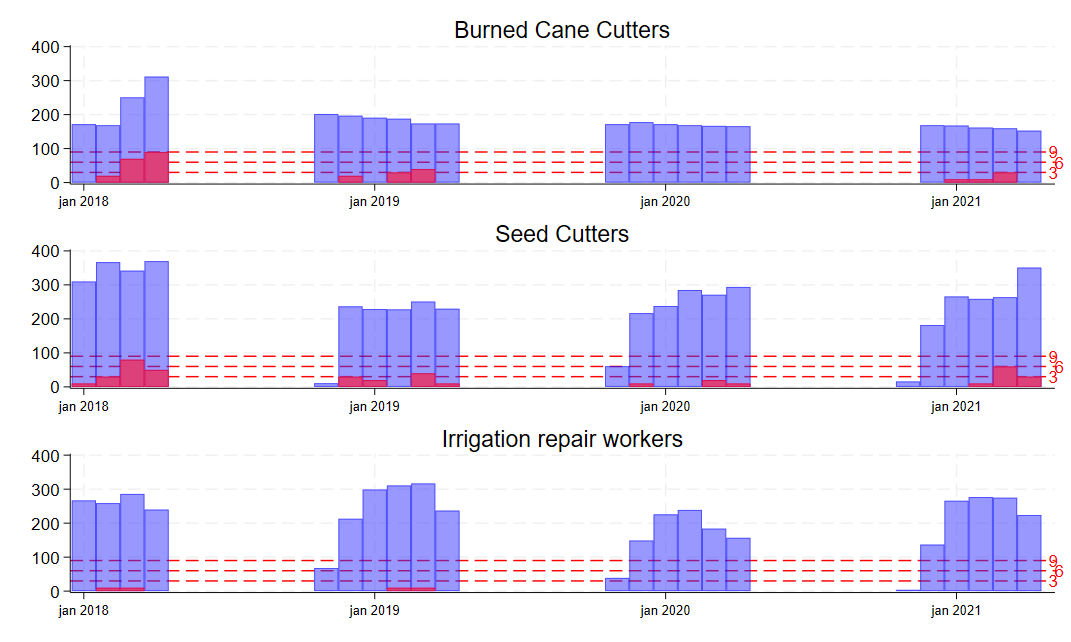


Supplement Figure 1. Clinical AKI cases (red) and number of workers (blue) per group and month during Ingenio San Antonio harvest seasons 2018-2021
